# Diabetic Ketoacidosis Presenting With New-Onset Generalized Tonic–Clonic Seizures and Status Epilepticus in a Previously Undiagnosed Diabetic Patient: A Case Report

**DOI:** 10.7759/cureus.100794

**Published:** 2026-01-05

**Authors:** Fatima Aziouaz, Dalia Kaadan, Wiam Ftouh, Yousra Dakkon, Mariem Benkacem

**Affiliations:** 1 Endocrinology, Diabetology and Metabolic Diseases Department, Tangier University Hospital, Tangier, MAR; 2 Faculty of Medicine and Pharmacy of Tangier, Faculty of Medicine and Pharmacy of TangierAbdelmalek Essaâdi University, Tangier, MAR; 3 Faculty of Medicine and Pharmacy of Tangier, Abdelmalek Essaâdi University, Tangier, MAR

**Keywords:** diabetic ketoacidosis, latent autoimmune diabetes in adults, metabolic encephalopathy, neurological complications, new-onset seizures, status epilepticus

## Abstract

We present the case of a 47-year-old Moroccan male with no known history of diabetes who presented with new-onset status epilepticus. His family reported a one-year history of increasing lethargy, polyuria, polydipsia, significant weight loss, and polyphagia. Initial laboratory investigations revealed severe hyperglycemia (547 mg/dL), marked ketosis, and profound metabolic acidosis (bicarbonate level of 9 mmol/L), confirming the diagnosis of severe diabetic ketoacidosis. Further investigations, including an elevated HbA1c level (15.2%), positive anti-glutamic acid decarboxylase (GAD) antibodies (280 IU/mL), and a low fasting C-peptide level (0.06 ng/mL), supported the diagnosis of new-onset latent autoimmune diabetes in adults. Neuroimaging and electroencephalography ruled out other neurological etiologies, supporting diabetic ketoacidosis-induced status epilepticus as the most likely cause. Status epilepticus as the initial manifestation of diabetic ketoacidosis secondary to latent autoimmune diabetes in adults is rare and clinically challenging. Treatment consisted of intravenous fluids, regular insulin, and phenobarbital for seizure control, leading to progressive clinical and biochemical improvement. This case highlights the importance of early metabolic evaluation in adults presenting with new-onset seizures, even in the absence of previously known diabetes, to allow timely diagnosis and prevent severe neurological complications.

## Introduction

Diabetic ketoacidosis is a severe and potentially life-threatening complication of uncontrolled diabetes mellitus, characterized by hyperglycemia, metabolic acidosis, and ketonemia [1]. It may serve as the initial presentation of diabetes, particularly in previously undiagnosed individuals [2]. When diabetic ketoacidosis manifests with neurological symptoms, such as seizures or altered mental status, it poses significant diagnostic challenges, especially in patients without a known history of diabetes [3].

Latent autoimmune diabetes in adults is a slowly progressive autoimmune form of diabetes that occurs in adulthood and is frequently misclassified as type 2 diabetes because of its more indolent course. Although usually characterized by gradual β-cell failure, it may occasionally present with acute metabolic decompensation, including diabetic ketoacidosis.

We report the case of a patient presenting with new-onset seizures ultimately attributed to diabetic ketoacidosis, highlighting the importance of considering metabolic derangements in the differential diagnosis of neurological emergencies [4]. This case report underscores the necessity of a comprehensive metabolic evaluation in patients presenting with unprovoked seizures, even in the absence of a known history of diabetes [5]. Such atypical presentations, although less frequent than classic diabetic emergencies, may lead to diagnostic delays and increased morbidity if the underlying metabolic disturbance is not promptly recognized and treated [4]. Furthermore, this case emphasizes that status epilepticus may be the initial manifestation of diabetic ketoacidosis secondary to latent autoimmune diabetes in adults, underscoring the need for early metabolic evaluation in adults presenting with new-onset seizures [6].

## Case presentation

A 47-year-old Moroccan male with a medical history significant only for smoking and drug abuse presented to the emergency department after experiencing status epilepticus lasting approximately 30 minutes. His family reported a one-year history of progressive lethargy, polyuria, and polydipsia, associated with significant weight loss and polyphagia.

Upon arrival, the patient was obtunded and exhibited generalized tonic-clonic movements, prompting immediate medical intervention, including airway management. Anticonvulsant therapy with intravenous phenobarbital was administered for 24 hours. Initial laboratory investigations revealed severe hyperglycemia, marked ketonuria, and profound metabolic acidosis. The laboratory parameters confirming the diagnosis of severe diabetic ketoacidosis are summarized in Table 1.

**Table 1:** Initial laboratory findings at admission confirming severe diabetic ketoacidosis

Parameter	Obtained Value	Reference range
Capillary blood glucose	500 mg/dL	70–110 mg/dL
Urinary ketones (dipstick)	+4	Negative
Serum bicarbonate (HCO_3_^−^)	9 mmol/L	22–28 mmol/L

**Figure 1:**
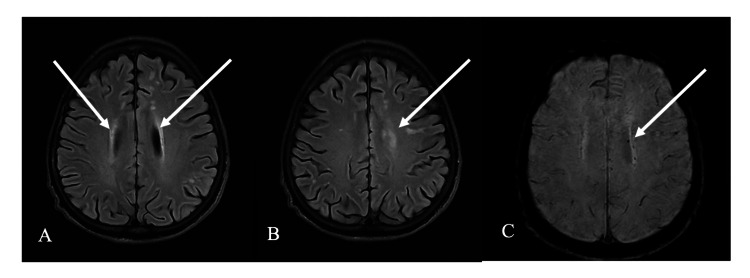
Brain MRI findings consistent with diabetic ketoacidosis–related metabolic encephalopathy, with alternative causes of white-matter disease considered less likely given the acute metabolic context and ancillary investigations. (A, B) Axial FLAIR MRI showing bilateral hyperintense lesions in the periventricular and subcortical white matter (arrows). (C) Axial susceptibility-weighted imaging demonstrating punctate microbleeds in the periventricular region (arrow).

After seizure control, the patient’s neurological status improved, and he regained consciousness without focal neurological deficits, although postictal confusion persisted for several hours. Brain computed tomography revealed no acute structural abnormalities. Brain magnetic resonance imaging showed multiple white-matter abnormalities, hyperintense on T2-weighted and fluid-attenuated inversion recovery (FLAIR) sequences in the subcortical and periventricular regions, with associated microbleeds on susceptibility-weighted imaging; these findings were consistent with metabolic encephalopathy in the given clinical context (Figure 1).

An electroencephalogram performed after seizure control demonstrated a normal background rhythm without paroxysmal abnormalities or epileptiform discharges. Cerebrospinal fluid analysis revealed elevated glucose levels (2.88 g/L), with normal cell counts and negative cultures. Metabolic encephalopathy secondary to diabetic ketoacidosis was therefore considered the most likely etiology of the patient’s neurological presentation, consistent with previous reports describing a wide spectrum of neurological manifestations associated with diabetic ketoacidosis (DKA), ranging from altered consciousness to severe and refractory seizures [3].

This case highlights the importance of considering metabolic disturbances such as diabetic ketoacidosis in the differential diagnosis of new-onset seizures, particularly in patients presenting with suggestive clinical features such as unexplained weight loss and polyuria [3]. There was no family history of epilepsy or diabetes mellitus, supporting a secondary cause of diabetes.

Further evaluation, including a psychiatric assessment, was conducted to determine whether drug addiction or abrupt cessation could have precipitated the status epilepticus as part of a withdrawal syndrome. The assessment, confirmed by the psychologist at the University Hospital of Tangier, revealed no evidence of withdrawal. The patient returned to a normal level of consciousness within 24 hours, without aggressive behavior, seizure recurrence, or the need for additional antiepileptic treatment.

Further laboratory evaluation revealed glycated hemoglobin (HbA1c) markedly elevated at 15.2%, positive anti-glutamic acid decarboxylase (anti-GAD) antibodies (280 IU/mL), and markedly reduced fasting C-peptide level (0.06 ng/mL), findings consistent with latent autoimmune diabetes in adults. An autoimmune workup performed after clinical stabilization showed normal morning cortisol, thyroid-stimulating hormone, and anti-tissue transglutaminase IgA and IgG antibodies, excluding associated autoimmune adrenal or thyroid disorders. Additional laboratory investigations performed during hospitalization are summarized in Table 2.

**Table 2:** Extended laboratory investigations performed during hospitalization. VDRL: Venereal Disease Research Laboratory; TPHA: Treponema Pallidum Hemagglutination

Parameter	Obtained Value	Reference range
Serum bicarbonate (HCO₃⁻)	9 mmol/L	22–28 mmol/L
White blood cells (WBC)	6.69 ×10³/µL	4.0–10.0 ×10³/µL
Red blood cells (RBC)	4.29 ×10⁶/µL	4.5–5.9 ×10⁶/µL
Hemoglobin (Hb)	14 g/dL	13–17 g/dL
Hematocrit (Hct)	41.3 %	40–52 %
Mean corpuscular hemoglobin (MCH)	32.7 pg	27–33 pg
Mean corpuscular hemoglobin concentration (MCHC)	34 g/dL	32–36 g/dL
Platelets (PLT)	376 ×10³/µL	150–400 ×10³/µL
International normalized ratio (INR)	1.5	0.9–1.2
Glycated hemoglobin (HbA1c)	15.2 %	< 5.7 %
Thyroid-stimulating hormone (TSH)	1.65 µIU/mL	0.35–4.94 µIU/mL
Morning serum cortisol (8 a.m.)	23.20 µg/dL	4.8–26.2 µg/dL
Anti-glutamic acid decarboxylase antibodies (anti-GAD)	280 IU/mL	<17 IU/mL
Alanine aminotransferase (ALT)	9 U/L	7–55 U/L
Aspartate aminotransferase (AST)	21 U/L	8–48 U/L
Serum creatinine	4.8 mg/L	6–13 mg/L
Blood urea	0.13 g/L	0.15–0.45 g/L
Serum sodium (Na⁺)	130 mmol/L	135–145 mmol/L
Serum potassium (K⁺)	3.8 mmol/L	3.5–5.1 mmol/L
Serum chloride (Cl⁻)	104 mmol/L	98–107 mmol/L
Serum phosphorus	33 mg/L	25–45 mg/L
Total calcium	77 mg/L	85–105 mg/L
Serum albumin	28 g/L	35–50 g/L
Corrected calcium	90 mg/L	85–105 mg/L
C-reactive protein (CRP)	84 mg/L	< 5 mg/L
Procalcitonin	0.09 ng/mL	< 0.1 ng/mL
Hepatitis B serology	Negative	Negative
Hepatitis C serology	Negative	Negative
Syphilis serology (VDRL, TPHA)	Negative	Negative

Treatment focused on acute metabolic stabilization and long-term glycemic control. Intravenous regular insulin and fluid resuscitation were initiated to correct hyperglycemia, acidosis, and electrolyte disturbances. To control the status epilepticus, phenobarbital was administered by the neurology team as an intravenous loading dose of 15 mg/kg, followed by a maintenance dose of 5 mg/kg for 24 hours, after which it was discontinued following complete clinical resolution of seizures.

After stabilization, the patient was transitioned to a basal-bolus insulin regimen, which is appropriate for the management of latent autoimmune diabetes in adults (LADA) given its autoimmune etiology and progressive beta-cell failure [7]. Glycemic control was closely monitored, with progressive resolution of ketosis and metabolic abnormalities [8]. This comprehensive therapeutic approach addressed both the acute life-threatening manifestations and the long-term management of diabetes, aiming to prevent recurrence and future complications [9]. Subsequent follow-up demonstrated improved glycemic control, with decreasing HbA1c levels.

## Discussion

Diabetic ketoacidosis is a severe acute metabolic complication of diabetes mellitus, characterized by hyperglycemia, ketosis, and metabolic acidosis resulting from absolute or relative insulin deficiency [10]. This deficiency promotes unregulated lipolysis, leading to excessive ketone body production that overwhelms the body’s buffering capacity and results in a profound acidotic state [11]. Neurological manifestations, such as status epilepticus as observed in this case, are uncommon but represent serious complications, often related to severe metabolic disturbances and osmotic shifts that adversely affect cerebral function [3]. Although rare, these neurological complications require prompt recognition and aggressive management to reduce the risk of irreversible brain injury and mortality [12]. In the present case, new-onset latent autoimmune diabetes in adults revealed by diabetic ketoacidosis complicated by status epilepticus highlights the broad clinical spectrum of diabetes and the importance of recognizing atypical presentations, even in relatively young individuals [9].

Pathophysiology of diabetic ketoacidosis and seizures

The profound metabolic acidosis and electrolyte imbalances characteristic of diabetic ketoacidosis can significantly alter neuronal excitability, thereby lowering the seizure threshold and precipitating epileptic activity [13]. Hyperglycemia-induced osmotic shifts may promote cerebral edema, while electrolyte disturbances such as hyponatremia or hypophosphatemia further impair neuronal function [14]. In addition, the accumulation of ketone bodies, particularly β-hydroxybutyrate and acetoacetate, may exert neurotoxic effects or disrupt neurotransmitter homeostasis, contributing to seizure generation [4].

The interaction of these mechanisms creates a highly proconvulsant cerebral environment during diabetic ketoacidosis [15], underscoring the importance of rapid metabolic correction to prevent or mitigate neurological sequelae [16]. Although ketone bodies are generally considered to have anticonvulsant properties, rapid fluctuations in ketone levels during DKA treatment, combined with abrupt osmotic changes, may paradoxically trigger or exacerbate seizure activity [4]. This phenomenon may involve endothelial activation and increased expression of adhesion molecules such as intercellular adhesion molecule-1 in cerebrovascular endothelial cells, contributing to blood-brain barrier dysfunction and cerebral edema [17].

Moreover, overly rapid correction of hyperglycemia may induce a sudden decrease in serum osmolality, increasing the risk of cerebral edema, a rare but potentially fatal complication, particularly in pediatric populations [3,18,19]. This highlights the delicate balance required in managing diabetic ketoacidosis, where insulin and fluid therapy must be carefully titrated to avoid iatrogenic complications such as cerebral edema or osmotic demyelination syndrome [3]. The resulting cerebral edema or metabolic encephalopathy can lead to diffuse cerebral dysfunction even in the absence of structural brain lesions [3].

Differential diagnosis

In patients with diabetic ketoacidosis and neurological manifestations, differentiating true status epilepticus from conditions such as metabolic encephalopathy, nonconvulsive status epilepticus, or cerebral edema is essential for appropriate management. Clinical features may overlap, making diagnosis challenging without a comprehensive evaluation that includes neuroimaging and electroencephalography [20].

Early recognition of cerebral edema, a leading cause of neurological morbidity and mortality in pediatric diabetic ketoacidosis, is particularly difficult but critical [21,22]. Early warning signs include headache, irritability, bradycardia, and progressive alterations in mental status [23]. Prompt recognition and intervention are vital, as cerebral edema remains the most severe complication of diabetic ketoacidosis in children and adolescents [23]. Although rare in adults, this complication carries a reported mortality rate of up to 40-90% in pediatric cases, necessitating rapid and precise therapeutic intervention [24].

Treatment considerations

The cornerstone of diabetic ketoacidosis management consists of controlled fluid resuscitation, insulin therapy, and meticulous electrolyte correction, all carefully titrated to prevent rapid osmotic shifts that could exacerbate cerebral edema [23,25,26]. However, the optimal fluid resuscitation strategy remains debated, with ongoing research refining guidelines to minimize neurological complications while ensuring effective metabolic correction [23].

Conversely, excessively rapid correction of hyperglycemia and acidosis without close monitoring may paradoxically result in neurological deterioration, including seizures, despite apparent metabolic improvement [27]. This emphasizes the delicate balance required in DKA management, particularly regarding fluid administration rates, which, if not carefully controlled, may increase the risk of neurological complications [28].

Clinical implications

A wide range of neurological complications has been described in association with diabetic ketoacidosis, including cerebral edema with increased intracranial pressure leading to coma, focal or generalized seizures, and cerebrovascular events resulting in motor or sensory deficits [29]. Although heterogeneous in presentation, these complications collectively underscore the importance of vigilant neurological monitoring and aggressive metabolic management to prevent long-term sequelae and improve patient outcomes [30]. This case also highlights the need for early recognition of metabolic causes in adults presenting with new-onset seizures or status epilepticus. Prompt metabolic evaluation is essential, as rapid identification and correction of diabetic ketoacidosis may prevent irreversible neurological injury. In addition, latent autoimmune diabetes in adults should be considered in non-obese adults presenting with diabetic ketoacidosis, particularly when autoimmune markers are positive or the C-peptide level is low. Greater awareness of this entity may help avoid diagnostic delay and ensure appropriate long-term management.

## Conclusions

Status epilepticus represents a rare but potentially life-threatening manifestation of severe metabolic disturbances such as diabetic ketoacidosis. This case highlights the importance of early recognition and prompt treatment of hyperglycemic emergencies in order to reduce the risk of acute neurological complications. It also underscores the role of healthcare professionals in patient education regarding diabetes, its warning signs, and its acute complications. Reinforcing adherence to insulin therapy, dietary measures, and regular glucose monitoring is important to help prevent recurrence. Although the strength of the conclusions is inherently limited by the descriptive nature of a single case report, this observation emphasizes the need for clinical vigilance and multidisciplinary care in similar presentations.

## References

[1] Meaden CW, Kushner BJ, Barnes S (2018). A rare and lethal complication: Cerebral edema in the adult patient with diabetic ketoacidosis. Case Rep Emerg Med.

[2] Menakuru SR, Priscu A, Dhillon VS, Salih A (2022). Diabetic ketoacidosis as the initial presenting symptom of pancreatic adenocarcinoma: A discussion about screening utilizing ENDPAC Scoring coupled with CT scans and endoscopic ultrasound. Case Rep Oncol.

[3] Tomkins M, McCormack R, O'Connell K, Agha A, Merwick Á (2019). Metabolic encephalopathy secondary to diabetic ketoacidosis: A case report. BMC Endocr Disord.

[4] Bartolini E, Valenti R, Sander JW (2022). Hyperosmolar hyperglycaemic state causing atypical status epilepticus with hippocampal involvement. Pract Neurol.

[5] Thu WM, Zaw HH (2023). Hyperglycaemia-related visual seizure. Eur J Case Rep Intern Med.

[6] Meshref M, Hewila IM, Abdel Mageed S, Morra ME (2021). COVID-19 associated with encephalitis: Case report and review of literature. The Neurologist.

[7] Dentali F (2024). XXIX Congresso Nazionale della Società Scientifica FADOI | 11-13 maggio 2024. Italian J Med.

[8] Xenou M, Zoupas I, Lygnos D, Fousteris E (2022). Diabetic ketoacidosis as first presentation of latent autoimmune diabetes in adults in a patient with hashitoxicosis as first presentation of Hashimoto's thyroiditis: A case report. J Med Case Rep.

[9] Rahmadi A, Decroli E, Kam A (2019). Sepsis in latent autoimmune diabetes in adults with diabetic ketoacidosis: A case report. Open Access Maced J Med Sci.

[10] Vadasz B, Arazi M, Shukha Y, Koren O, Taher R (2021). Sodium-glucose cotransporter-2-induced euglycemic diabetic ketoacidosis unmasks latent autoimmune diabetes in a patient misdiagnosed with type 2 diabetes mellitus: A case report. J Med Case Rep.

[11] Özdemir B, Erismis B, Kocoglu H (2016). Is diabetes mellitus complicated by ketoacidosis in the elderly always latent autoimmune diabetes of the adult?. J Diabetes Metab Disord Control.

[12] Oliveira DA, Arca VM, de Holanda AC (2023). Bilateral pallido-nigral lesions in a patient with subacute chorea after diabetic ketoacidosis: Case-report. Sao Paulo Med J.

[13] Darii F, Romaniuc D, Иванов BB, Porcereanu N, Tagadiuc O (2025). Euglycemic diabetic ketoacidosis in the context of latent autoimmune diabetes and SGLT2 inhibitor therapy: Case report. Arch Balk Med Union.

[14] Yoon JS, Park KJ, Sohn YB, Lee HS, Hwang JS (2018). Successful switching from insulin to sulfonylurea in a 3-month-old infant with diabetes due to p.G53D mutation in KCNJ11. Ann Pediatr Endocrinol Metab.

[15] (2025). 51st Annual Conference of the International Society for Pediatric and Adolescent Diabetes (ISPAD): Abstracts. Horm Res Paediatr.

[16] Kaya FB, ÇEVİK AA, Kaya Ş, OZAKIN E, Acar N (2014). Is it really diabetic ketoacidosis? Double trouble. DergiPark (Istanbul University).

[17] Kanikarla-Marie P, Jain SK (2016). Hyperketonemia and ketosis increase the risk of complications in type 1 diabetes. Free Radic Biol Med.

[18] Mozzillo E, D'Amico A, Fattorusso V (2015). Cerebral accidents in pediatric diabetic ketoacidosis: Different complications and different evolutions. Horm Res Paediatr.

[19] Kabashneh S, Al-Sagri Z, Alkassis S, Shanah L, Ali H (2020). Diabetic ketoacidosis complicated by a brain death. Cureus.

[20] Lim YX, Kwek SY, How CH, Chan WS (2020). A clinical approach to encephalopathy in children. Singapore Med J.

[21] Magomedova KS, Bykov YV, Baturin VA (2023). Diabetic ketoacidosis and cognitive impairment in children and adolescents. (Article in Russian). Bull Siberian Med.

[22] Williams V, Mohandoss V (2021). Portending complications in pediatric diabetic ketoacidosis. Indian J Crit Care Med.

[23] Alsabri M, Rath S, Okaruefe CO (2025). Diabetic ketoacidosis in pediatric emergency medicine: Risk factors, myths, and evidence-based management of complications. Curr Emerg Hospital Med Rep.

[24] Umpierrez GE, Murphy MB, Kitabchi AE (2002). Diabetic ketoacidosis and hyperglycemic hyperosmolar syndrome. Diabetes Spectr.

[25] Nunes KP (2015). Major topics in type 1 diabetes.

[26] Ishii M (2017). Endocrine emergencies with neurologic manifestations. Continuum (Minneap Minn).

[27] Glaser N, Yuen N, Anderson SE, Tancredi DJ, O'Donnell ME (2010). Cerebral metabolic alterations in rats with diabetic ketoacidosis: Effects of treatment with insulin and intravenous fluids and effects of bumetanide. Diabetes.

[28] Wolfsdorf J, Glaser N, Sperling MA (2006). Diabetic ketoacidosis in infants, children, and adolescents: A consensus statement from the American Diabetes Association. Diabetes Care.

[29] Kavathia S, Kataria S, Patel N, Patel S (2023). Diabetic ketoacidosis-induced "terrible triad" associated with seizures and acute renal failure: A report of a rare case. Cureus.

[30] Azova S, Rapaport R, Wolfsdorf J (2021). Brain injury in children with diabetic ketoacidosis: Review of the literature and a proposed pathophysiologic pathway for the development of cerebral edema. Pediatr Diabetes.

